# When It Is Not NEC: Recognizing Mimics of Necrotizing Enterocolitis in Preterm Infants

**DOI:** 10.3390/children13081022

**Published:** 2026-07-31

**Authors:** Dwayne Mascarenhas, Bonny Jasani

**Affiliations:** 1 Department of Pediatrics, Mount Sinai Hospital, Toronto, ON M5G 1X8, Canada; dwayne.mascarenhas@sinaihealth.ca; 2 Department of Pediatrics, University of Toronto, Toronto, ON M5S 1A1, Canada; 3 Division of Neonatology, The Hospital for Sick Children, 555 University Avenue, Toronto, ON M5G 1X8, Canada

**Keywords:** necrotizing enterocolitis, preterm infant, differential diagnosis, spontaneous intestinal perforation, neonate, misdiagnosis

## Abstract

Necrotizing enterocolitis (NEC) is a major cause of morbidity and mortality in preterm infants, yet it remains one of the most challenging neonatal diagnoses to establish with certainty. No single clinical, laboratory, or radiographic finding is pathognomonic, and the modified Bell’s staging system is limited because features such as abdominal distension, bloody stools, pneumatosis intestinalis, and portal venous gas are common in preterm infants or are mimicked by other conditions. A wide range of disorders in preterm and term neonates can therefore resemble NEC, including spontaneous intestinal perforation, sepsis-associated ileus, dysmotility of prematurity, malrotation with or without volvulus, cow’s milk protein allergy, Hirschsprung-associated enterocolitis, cardiogenic colitis, viral enterocolitis, intussusception, neonatal appendicitis, meconium obstruction, incarcerated inguinal hernia, and food protein-induced enterocolitis syndrome. Misclassification of these mimics leads to over- and under-estimation of NEC incidence, contaminates outcome data, and may delay surgical intervention. This review summarizes the epidemiology, risk factors, pathophysiology, and distinguishing clinical, radiographic, and histopathological features of the common mimics of NEC, with particular emphasis on spontaneous intestinal perforation. We also highlight the inherent diagnostic challenges and future directions, including the need for reliable biomarkers and the evolving role of artificial intelligence.

## 1. Introduction

Necrotizing enterocolitis (NEC) is a devastating gastrointestinal complication in very-low-birth-weight (VLBW) neonates, with an incidence of ~7% [1]. Surgical NEC comprises approximately 40% of these infants, with an overall incidence of ~3% [2]. Surgical NEC has been associated with significant neonatal morbidity and mortality, with large epidemiologic studies reporting a mortality rate of 23–50% in surgical NEC [3,4,5,6]. Even beyond the neonatal period, surgical NEC continues to be associated with poor outcomes across all domains [7,8,9]. A recent systematic review of 60,346 infants showed that preterm infants with NEC had 1.9 times increased odds of having neurodevelopmental impairment after adjusting for confounders. Additionally, they were also at higher risk of severe intraventricular hemorrhage [Odds Ratio (OR) 1.42] and periventricular leukomalacia (OR 2.55). Compared to medical NEC, infants with surgical NEC had an even higher risk of neuro-developmental impairment (OR 1.78) and severe intraventricular hemorrhage (OR 1.57) [10]. In a study by Okten et al., extreme prematurity (<28 weeks’ gestation), lower birth weight, use of peritoneal drains, and enterostomies were identified as risk factors that could adversely affect the neurodevelopmental outcomes in these infants [11].

Although NEC can occur at any time in the neonatal period, it is commonly seen around 31–33 weeks postmenstrual age (PMA) [12,13]. A retrospective cohort study by Gordon et al. reported an increase in median postnatal age at diagnosis as gestational age (GA) decreases, with spontaneous intestinal perforation and surgical NEC being diagnosed earlier than medical NEC. Additionally, the median PMA at diagnosis was also noted to increase with advancing GA [14].

Diagnosing NEC has been a clinical challenge due to the absence of a single pathognomonic sign or diagnostic test. Traditional staging systems have been used in the past to grade the severity of NEC, the most common being the Walsh and Kliegman modification of Bell’s staging (Appendix A) [15,16]. However, there are several limitations with this staging system as well. Clinical signs such as abdominal distension are very common in the preterm population due to weak abdominal musculature. Abdominal distension is further worsened by poor intestinal motility and non-invasive ventilation. Other signs in Stage 1, such as blood in stools, may be seen with anal fissures, traumatic glycerine suppository insertion, volvulus without malrotation (VWM), as well as with other conditions such as Hirschsprung-associated enterocolitis (HAEC), cow’s milk protein allergy (CMPA), and food protein-induced enterocolitis syndrome (FPIES). This has led large studies to consider only Bell’s stage 2 and above (definite and advanced NEC).

Radiological signs of NEC may also be misleading as pneumatosis intestinalis (PI) is often mistaken for intraluminal gas that is mixed with stools, and portal venous gas (PVG) may be noted in preterm infants with umbilical venous catheters. Additionally, the sensitivity and specificity of radiographs and ultrasound in identifying these radiological signs have been found to vary significantly based on technology available and operator experience. While the finding of pneumoperitoneum is typical of stage IIIB NEC, a separate entity called spontaneous intestinal perforation, seen in extremely-low-birth-weight (ELBW) infants, was subsequently identified. Furthermore, the clinical course of NEC does not always conform to the stepwise progression outlined in Bell’s staging system; severe disease may develop in the absence of preceding characteristic clinical or radiological findings, thereby limiting opportunities for early surgical intervention.

While NEC is classically a disease of the premature gut, a similar clinical picture has been seen in both preterm and term neonates with intestinal anomalies, including intestinal atresia and Hirschsprung disease, as well as those with impaired mesenteric perfusion such as critical congenital heart disease, maternal cocaine use, and perinatal asphyxia. This highlights a significant overlap in the clinical presentation of NEC and other differential diagnoses. Misclassification of NEC results in both over- and under-estimation of the incidence of NEC in the preterm population [17].

A comprehensive literature search of PubMed, EMBASE, CINAHL, and the Cochrane databases was conducted from 1 January 1966 to 1 July 2026, using terms related to necrotizing enterocolitis and its differential diagnoses, to identify relevant articles for this review.

## 2. Differential Diagnoses

Given the non-specific nature of many clinical, laboratory, and radiographic findings associated with NEC, a broad differential diagnosis should be considered in infants presenting with features suggestive of the disease. Key differential diagnoses and their specific risk factors are summarized in Table 1 and Table 2.

### 2.1. Spontaneous Intestinal Perforation (SIP)

SIP, also referred to as focal intestinal perforation, is a surgical emergency characterized by a focal perforation in the intestine, most commonly occurring in the antimesenteric border of the distal ileum [66]. The incidence of SIP in preterm infants has been reported to vary between 2% in VLBW (<1500 g birth weight) infants [67], and 8% in ELBW (<1000 g birth weight) infants [68]. When stratified by gestational age, around 1% of infants below 32 weeks GA develop SIP [69], with a higher incidence of 4% in periviable infants <25 weeks GA [20].

This condition occurs predominantly in extremely preterm infants with increasing incidence with decreasing gestational age [18,19,20]. A study from the US National Inpatient Sample dataset, including over 10,000 infants with SIP, reported that 82% were ELBW and 90% were ≤28 weeks of gestation, and were more likely to be male [70]. In contrast to NEC, which usually presents around 31–33 weeks PMA, SIP typically occurs in the first 1–2 weeks after birth [18,19]. However, the timing of presentation alone cannot be used to determine between NEC and SIP as there is an overlap with early NEC and late-presenting SIP [71]. The earlier onset of SIP compared with NEC (median age at presentation: 7 vs. 15 days), together with its higher incidence among infants of lower gestational age and birth weight, may aid in distinguishing SIP from NEC [26,66,72].

#### 2.1.1. Risk Factors

Prematurity is one of the strongest risk factors for the development of SIP, particularly in the extremely preterm population. The mechanisms underlying the different risk factors are described in Table 3.

A contrasting feature between SIP and NEC is the history of feeding. Unlike infants with NEC, who are being fed a substantial amount of feed volume, with or without fortification, infants with SIP may have received no, or minimal, feed volumes prior to perforation [18,22]. In ELBW infants, early enteral feeding was associated with a reduction in the incidence of SIP [73]. Clinically, SIP has been associated with concurrent sepsis with systemic candidiasis [31].

#### 2.1.2. Pathophysiology

SIP is hypothesized to be caused by changes in the vessel wall, particularly in ELBW infants. Steroids have been shown to cause changes to the intestinal wall resulting in mucosal hyperplasia and submucosal thinning [22]. A mouse model demonstrated that the concurrent use of steroids and indomethacin reduced all three forms of intestinal nitric oxide synthetase (NOS) resulting in reduced spontaneous peristalsis, and thereby increased vulnerability of the ileum of the neonatal mouse to perforation [74]. Steroids interfere with the neuronal NOS protein, while indomethacin reduces the function of endothelial NOS, together resulting in a near-total loss of NOS in the ileum. Additionally, there is a concomitant loss of smooth muscle with impaired distal intestinal motility [74].

#### 2.1.3. Clinical Presentation

Neonates with SIP most commonly present in the first 2 weeks of life with acute onset of abdominal distension, frequently associated with bluish discoloration of the abdomen [66]. The bluish discoloration of the abdomen in SIP is due to the peritoneal staining by leaked blood, meconium, and bacteria, unlike in NEC, where there is more commonly abdominal wall erythema due to the spreading inflammatory process upward along the fascial planes to the subcutaneous and cutaneous layers [75]. Bloody stools, seen due to mucosal necrosis and sloughing, are typically absent in SIP. Infants with SIP appear less sick, and the systemic signs characteristically associated with NEC such as metabolic acidosis, hyponatremia, thrombocytopenia, and shock are less commonly seen. However, these may develop subsequently as SIP progresses to peritonitis.

#### 2.1.4. Investigations

X-ray findings include free air in the abdomen in the absence of other features of NEC such as PI and PVG, highlighting that SIP does not follow the progression of stages as described by Bell’s staging for NEC [15,16]. Though isolated free air is characteristic of SIP, a gasless abdomen is a more common finding on abdominal X-ray [76]. Findings on ultrasound of the abdomen include free intra-abdominal air and echogenic free fluid or complex ascites with normal bowel perfusion and thickness [76].

Histopathological examination of the resected bowel reveals a focal perforation and an area of hemorrhagic necrosis without the typical coagulation necrosis that is seen in NEC [66]. There is minimal inflammation and no evidence of ischemia, highlighting the focality of the pathology. Immunohistological staining of resected specimens using markers such as hypoxia inducible factor 1α and glucose transporter 1 reveals the primary role of ischemia in the pathogenesis of NEC and not SIP [77]. The mucosa is well preserved rather than necrotic, and the muscularis externa shows isolated areas of necrosis in around 30% of the cases. There may be an absence of the internal muscularis propria or muscularis mucosa noted in infants with SIP [78].

Taken together, several clinical and radiological characteristics may assist in distinguishing SIP from NEC, including lower GA and birth weight, earlier PMA at presentation, recent antenatal corticosteroid exposure (<7 days prior to delivery) in combination with early postnatal indomethacin administration, minimal enteral feeding, and the presence of a gasless abdomen or pneumoperitoneum without accompanying systemic inflammatory manifestations. Nevertheless, considerable overlap exists between the two entities, and a definitive diagnosis of SIP is established primarily through characteristic intraoperative gross findings and histopathological evaluation.

#### 2.1.5. Outcomes

Misclassification of SIP as stage 3 NEC as per Bell staging has led to contamination of data and inaccurate estimation of long-term outcomes [79]. Mortality related to SIP was found to be lower than that in NEC in various observational studies [68,80]. A large retrospective multicenter study by the Eunice Kennedy Shriver National Institute of Child Health and Human Development group reported that both SIP and NEC were associated with an increase in the risk of mortality or neurodevelopmental impairment with adjusted odds ratio (aOR) of 2.21 and 2.11, respectively. Neurodevelopmental impairment alone among survivors was also increased in both the SIP (aOR 2.17) and NEC groups (aOR 1.70) [80]. The German Neonatal Network reported a higher risk of intelligence quotient scores < 85 at school age [Relative risk (RR) 3.0] in the NEC cohort compared to controls, which was not seen in the SIP cohort (RR 1.0) [81]. Similarly, length of hospital stay and growth morbidities were reported to be similar between the two groups, with growth impairment persisting into the late-childhood period [82,83].

### 2.2. Sepsis-Associated Ileus

Sepsis-associated ileus refers to the temporary paralysis of the intestine due to a systemic bacterial infection. This leads to accumulation of intestinal contents resulting in clinical features of abdominal distension, feeding intolerance, and increased gastric residuals. Unlike NEC, where there is extensive inflammation and necrosis of the bowel, sepsis-associated ileus results in functional loss with no injury to the intestinal wall itself. Clinical examination reveals abdominal distension and hypoperistalsis. Discoloration of the abdomen is uncommon in sepsis-associated ileus. Abdominal radiography reveals distended bowel loops and air-fluid levels, with little to no gas in the rectum. Treatment involves bowel rest and parenteral nutrition, along with a complete course of intravenous antibiotic therapy.

### 2.3. Volvulus with Malrotation

While more common in term infants, volvulus with malrotation has also been reported in preterm infants [84]. Clinical manifestations in preterm infants include feeding intolerance, marked abdominal distension, bloody stools, and apnea. Unlike the classical bilious vomiting seen in term infants, preterm infants with malrotation and volvulus do not present with bilious emesis, highlighting the high index of suspicion required for diagnosis [85]. Examination findings commonly include abdominal discoloration, severe abdominal distension, which may be tender, along with systemic signs of hypotension, respiratory failure, and bradycardia. Abdominal radiographs may show evidence of dilated loops of bowel and may even have PI, though malrotation is also commonly diagnosed using an upper GI contrast study. Ultrasound assessment for malrotation includes evaluation of the SMA/SMV relationship and the course of the duodenum. Normally, the SMV lies to the right of the SMA, and the third portion of the duodenum passes posterior to the SMA. In malrotation, the SMA/SMV relationship may be inverted or abnormal, and D3 may be positioned anterior to the SMA or fail to cross normally—findings that are highly suggestive of malrotation. These sonographic markers are supported by the AJR Expert Panel narrative review on ultrasound diagnosis of midgut malrotation and volvulus [86]. A classic finding of volvulus on ultrasound is the whirlpool sign, which has been reported as early as the fetal period [87]. The diagnosis of intestinal volvulus with malrotation is often established intraoperatively during laparotomy performed for presumed NEC. Progression to hemodynamic instability, respiratory failure, significant small bowel loss, and even death may ensue within hours if left surgically uncorrected.

### 2.4. Volvulus Without Malrotation (VWM)

Volvulus without malrotation (VWM) in preterm infants is a rare but life-threatening surgical emergency where a segment or loop of bowel twists around its own mesenteric axis without any pre-existing congenital rotation abnormalities. This peculiar condition was noted to occur more often in preterm infants who were small for gestational age, suggesting the role of intestinal hypoperfusion in the pathogenesis of this condition [34]. The infants usually presented with an acute abdomen with sudden-onset abdominal distension, visible bowel loops, abdominal tenderness, increased gastric residuals, blood in stools, and abdominal wall erythema, mimicking NEC. Intermittent and repeated episodes of abdominal distension were noted as an early marker, which could prompt the suspicion of intermittent twisting of the bowel. The VWM was reported to occur in the first week of life or several weeks after birth (median postnatal age around 45 days) after an asymptomatic period, usually beyond the time frame of NEC [34,39]. Risk factors including aggressive pro-peristaltic maneuvers such as intensive abdominal massage and pelvic rotation, as well as non-invasive respiratory support (especially with higher positive end-expiratory pressures) have been hypothesized from observational studies to be associated with VWM and may contribute to the occurrence of late-onset VWM [34,38,39]. Kargl et al. described volvulus without malrotation in two groups, one which presented with meconium obstruction of prematurity and the other without, the latter group requiring bowel resection [36]. The volvulus was noted to occur most commonly in the ileum. On abdominal imaging, features of ileus are commonly seen. The classical whirlpool sign and corkscrew sign are not seen due to the absence of malposition [36]. Pneumatosis may also be noted and suggests progression to NEC, which may prompt conservative medical management and a resultant delay in surgical intervention [36]. Timing of surgery is crucial, as irreversible bowel ischemia usually occurs by 24 h post onset, resulting in loss of bowel [88]. Studies have reported GA at birth, birth weight, timing of surgery, and length of bowel resection as key predictors of patient outcomes [34,89]. Some cases of VWM have also been reported in the fetal period, with good outcomes following surgical intervention [90,91].

### 2.5. Dysmotility of Prematurity

Gastrointestinal maturation progresses throughout gestation with gradually improving intestinal motor function. This is responsible for the forward propulsion of enterally-delivered feeds. Hence, preterm infants <34 weeks GA are at a higher risk of delayed gastric emptying and intestinal transit that may lead to intestinal dysmotility [92]. This manifests clinically as abdominal distension, gastric residuals, and emesis [32]. ELBW infants, use of caffeine citrate, and formula feeding were reported as risk factors [32]. The immaturity of the intestinal motor system leads to delayed transit and subsequent bacterial overgrowth and gaseous distension of the abdomen [93]. Clinically, these infants may present with feed intolerance, increased gastric residuals, and abdominal distension.

### 2.6. Cow’s Milk Protein Allergy (CMPA)

CMPA is an IgE-mediated classic food reaction which results in an immediate allergic response to ingestion of the inciting food agent. In addition to being immediate, the response is also reproducible and readily diagnosed by detection of food-specific IgE, which contrasts with food protein-induced enterocolitis syndrome (FPIES), in which symptoms develop after a few hours and are not characterized by the presence of a specific IgE [94]. IgE-mediated CMPA has been reported to have an incidence of 0.5% [95]. CMPA is a rare condition in preterm infants, though it has been described in case reports. A systematic review of 25 case reports found that a majority of the preterm infants were exposed to bovine-based milk protein prior to the development of symptoms [96]. A family history of allergic disorders has been associated with a large proportion of infants with CMPA [40]. Chinese infants with an rs1800896 variant in the IL-10 gene were associated with a genetic predisposition to CMPA [41]. Delivery by caesarean section was also hypothesized to be a risk factor due to the lack of maternal flora, which helps establish a healthy gut microbiome [42]. The proteins casein (major protein in cow’s milk) and beta-lactoglobulin, which are highly allergenic and heat-resistant proteins, have been implicated in the pathophysiology of CMPA. CMPA commonly presents in preterm infants with bloody stools, emesis, and abdominal distension [96]. Abdominal films reveal bowel wall thickening, though pneumatosis is uncommon. CMPA has been noted in neonates with extensive bowel resection and subsequent short-bowel syndrome in addition to sensitization to latex and egg later in childhood [97].

### 2.7. Hirschsprung-Associated Enterocolitis (HAEC)

HAEC is the leading cause of mortality in infants with Hirschsprung disease (HD). A systematic review of 4147 cases of HD across 26 publications reported the prevalence of HD in preterm infants of 6% [98]. With increasing awareness and detection, the prevalence of HD in preterm infants between 2000 and 2013 ranged from 4 to 19.4%, while total colonic aganglionosis was reported in 13% of HD cases. The mean GA for HD in preterm infants was 34.5 ± 0.7 weeks, and the mean PNA at diagnosis varied from 18.3 days up to 3.9 months [98]. Abnormal enteric innervation in HD leads to impaired intestinal motility, which results in dysbiosis of the intestinal microbiome. Failure of the intestinal barrier to tackle this overwhelming bacterial overgrowth culminates in an enterocolitis-like clinical picture. Preterm infants with HD commonly present with abdominal distension (80%), delayed passage of meconium (57%), or bile-stained emesis (37%) [98]. HAEC may present before or after definitive surgical intervention as fever, lethargy, emesis, loose bloody stools, and marked abdominal distension. HAEC is commonly treated with intravenous fluids, bowel rest, antibiotic coverage, including anaerobic coverage with metronidazole, and rectal irrigations. Surgical interventions such as proximal diversion are warranted in case of failure to improve despite medical management, or exploratory laparotomy in case of perforation [99].

### 2.8. Congenital Heart Disease (CHD)-Associated Colitis (Cardiogenic NEC)

Congenital heart disease (CHD)-associated colitis, also referred to as cardiogenic NEC in the published literature, is a well-known complication of CHD. NEC has been reported in 3.4–5.1% of neonates with CHD, with an incidence of 6.3% in VLBW infants [49,100]. Overall, NEC was found to be higher in infants with CHD as compared to those with no CHD (6% vs. 0.9%) [100]. The pathophysiology of CHD-associated necrotizing enterocolitis involves various mechanisms that converge to cause NEC. Hemodynamic factors, such as impaired mesenteric flow due to the diastolic steal phenomenon in duct-dependent systemic circulation, low cardiac output states leading to tissue-level hypoperfusion, and abnormal systemic vasculature, contribute to ischemia at the intestinal level [48,101,102]. Additionally, gut factors including microbiome dysbiosis and feed characteristics such as high caloric value and lower rates of human milk feeding further increase the risk of NEC in infants with CHD [103,104]. Cyanotic heart disease, in particular, hypoplastic left heart syndrome and truncus arteriosus, was independently associated with NEC after adjusting for confounders [48]. Other important risk factors in these infants included prematurity, highest dose of prostaglandin > 50 ng/kg/min, and the presence of clinical shock [47,48]. On the other hand, cardiac catheterization, patent ductus arteriosus, left ventricular outflow tract obstruction, need for cardiac surgery, and aortic clamping were not found to be associated with the development of NEC [47]. The mortality of infants with CHD-associated necrotizing enterocolitis was higher than that of infants with classical NEC (38% vs. 27%) [100].

### 2.9. Viral Enterocolitis

Viral enterocolitis is an important differential diagnosis in preterm neonates with suspected NEC, as the two conditions can present with similar gastrointestinal manifestations. Several studies have reported an association between viral infections—including rotavirus, cytomegalovirus, norovirus, and astrovirus—and NEC, particularly among VLBW infants, although whether these viruses directly cause NEC or represent a distinct disease process remains uncertain [104,105]. Clinically, viral enterocolitis may present with feeding intolerance, abdominal distension, and bloody mucoid stools, making differentiation from NEC challenging. However, the presence of extraintestinal manifestations such as myocarditis, hepatitis, coagulopathy, or encephalopathy may favor a viral etiology, as these are uncommon in isolated NEC. Confirmation of viral enterocolitis is achieved by identifying the viral pathogen, typically using stool polymerase chain reaction testing. Management is primarily supportive and includes bowel rest, fluid and electrolyte management, and treatment of secondary bacterial infection when clinically indicated; surgical intervention is reserved for complications such as intestinal perforation or progressive bowel necrosis.

### 2.10. Intussusception

Intussusception in preterm neonates is an extremely rare condition with only 52 reported cases in the literature [105]. A male predominance was observed, with a male-to-female ratio of 2.7:1, and the mean age at presentation was 10.6 days. The most common clinical features include abdominal distension, bilious gastric residuals, and bloody stools. The characteristic triad of abdominal mass, red currant jelly stool, which is a marker of advanced bowel necrosis, and vomiting are uncommon in preterm infants [105]. This clinical triad was noted in only a third of the cases but overlaps with the clinical presentation of NEC often leading to misdiagnosis and a delay in treatment of around 10 days [106]. The classical findings included palpation of a sausage-shaped mass in the abdomen or the presence of a target sign on abdominal ultrasound. Ileo-ileal intussusception was the most frequent, and the lead point was most commonly a Meckel’s diverticulum [105]. Intrauterine fetal compromise [53], acquired cytomegalovirus infection [54], and a duplication cyst [55] are commonly associated with intussusception in preterm infants. Nearly half of the cases were complicated by perforation, with subsequent sepsis being the leading cause of death [105]. Characteristically, these infants require surgical intervention and may fail to respond to the conservative management of NEC. Air or contrast enemas are also commonly used, both as a diagnostic and therapeutic test for intussusception. In pediatric cases, a radiologist may perform an air or liquid contrast enema, such as barium, under fluoroscopic guidance. This can confirm the diagnosis by outlining the intestines, and the pressure generated during the procedure can often reduce the intussusception without surgery [107]. Gentle pneumatic reduction has also been successfully tried in a preterm infant [108].

### 2.11. Neonatal Appendicitis

Appendicitis in neonates is another rare condition with an incidence of 0.04 to 0.2% and has been reported even in preterm neonates occasionally [57,109,110]. Male infants tend to be more affected than females [57,58]. Theories around etiological associations with Hirschsprung disease, obstruction associated with cystic fibrosis, and neonatal appendicitis as part of a localized NEC remain unproven with little scientific evidence [59,111]. Several risk factors commonly associated with NEC, including perinatal asphyxia, prolonged rupture of membranes, chorioamnionitis, congenital heart disease, and maternal smoking, have also been reported in cases of neonatal appendicitis, lending support to the hypothesis of a shared or overlapping pathophysiological mechanism [58]. An inflamed appendix within an inguinal hernia sac (Amyand’s hernia) represents around one-third of all cases of neonatal appendicitis [112]. The common presentation involves temperature instability, poor feeding, emesis, and abdominal distension [58,113]. Abdominal wall erythema, tenderness, palpable mass, or free air localized to the right iliac fossa should prompt the suspicion of neonatal appendicitis [58]. Abnormal laboratory investigations include elevated white blood cell count and C-reactive protein. Diagnosis may be made pre-operatively with ultrasound or even a CT scan, given that the benefits of diagnosis likely outweigh the risk of radiation-related cancer [113]. However, a definitive diagnosis of neonatal appendicitis can be made only intra-operatively, on histopathological examination of the resected surgical specimen, or during post-mortem [58]. Though initially mistaken for NEC, surgical intervention is commonly performed for worsening clinical NEC despite medical management leading to an intraoperative diagnosis of neonatal appendicitis [112]. About 80% of the cases are complicated by perforation, with an overall mortality of 23–34% [57,58,113].

### 2.12. Meconium Obstruction of Prematurity

Meconium obstruction of prematurity is a distinct clinical entity that has been increasingly recognized and reported among VLBW infants [60,61,62,63,64]. Risk factors include maternal hypertensive disorder (gestational or chronic hypertension), maternal treatment with magnesium sulphate, and fetal growth restriction [60,61,63]. Clinically, the neonates presented with abdominal distension at a mean PNA of 5.6 days. This was associated with passage of infrequent and/or small amounts of stool [63]. Abdominal radiographs showed multiple bowel loops with little or no air in the rectum. Contrast studies showed inspissated meconium as filling defects, most frequently in the distal ileum. Treatment with Gastrogriffin and N-acetylcysteine enemas has been reported to be both diagnostic and therapeutic [63]. Use of oral N-acetylcysteine has also been tried with promising benefit [61,62]. Failure to make a timely diagnosis could result in intestinal perforation [61,63].

### 2.13. Anal Fissure

Anal fissures in preterm neonates are small tears in the mucosal layer of the anal opening. They are regarded as the most common cause of rectal bleeding [114]. Anal fissures present as isolated scant bright blood streaks in the stool, unlike NEC where the blood is commonly admixed with the stool. These fissures are often caused by high-density caloric formula feeds or dehydration due to inadequate hydration leading to hard, difficult-to-pass stools. The infant may also have signs of discomfort while passing stools such as crying or flexing his or her legs. However, rectal bleeding may be mistakenly attributed to anal fissures, leading to a delay in the diagnosis and treatment of early stages of NEC [115]. Application of a thin barrier of petroleum jelly or zinc oxide cream to the perianal area may be helpful. Alternatively, polyethylene glycol 3350 may be used to help soften the stool and reduce ongoing mucosal trauma [116]. A rectal suction biopsy should be considered to rule out HD in resistant or recurrent cases.

### 2.14. Acute Mesenteric Ischemia (AMI)

Acute mesenteric ischemia (AMI) is a severe, life-threatening vascular emergency caused by abrupt obstruction to the superior and/or inferior mesenteric arteries, and has been reported in preterm infants [65]. The extent of bowel injury depends on the degree of arterial obstruction and is classified as occlusive and non-occlusive. Arterial thrombosis is the most common cause of the occlusive type, though the exact mechanism is not clearly understood. On the other hand, the non-occlusive type is hypothesized to be caused by a critical reduction in blood flow in the mesenteric vessels due to vasospasm of the arteries or poor circulatory state leading to gut ischemia. Both forms may present as an acute abdomen with distension, tenderness, discoloration, and emesis. Laboratory investigations commonly show metabolic acidosis with subsequent multi-organ dysfunction, in the absence of elevated C-reactive protein, thrombocytopenia, or blood culture positivity. Due to the overlapping symptoms with NEC, it is notoriously difficult to differentiate between the two. Ischemic involvement of the small bowel and mesentery that is restricted to the territory supplied by a mesenteric vessel is strongly suggestive of acute mesenteric ischemia. In comparison, NEC typically involves, but is not limited to, the terminal ileum, cecum, and proximal part of the ascending colon, which marks the watershed area between the mesenteric arteries. Additionally, NEC tends to present with more gradual deterioration as compared to AMI. Medical management involves aggressive fluid resuscitation, antibiotics, along with anticoagulation and vasodilators if diagnosed early.

### 2.15. Incarcerated Hernia

Incarcerated hernia is a critical differential diagnosis for NEC as both present with features of bowel obstruction, inflammation, and potential bowel ischemia. The hernia may occur internally in preterm infants through a defect in the mesentery [117,118] or through the inguinal canal [119,120]. NEC may also be seen as a complication of incarcerated inguinal hernia following surgical repair [121,122,123]. The bowel ischemia due to compromised bowel perfusion is multifactorial in NEC (inflammation, sepsis), whereas it is mechanical in an incarcerated hernia due to strangulation. Infants may present with feeding intolerance, abdominal distension, abdominal erythema, bilious vomiting, and bloody stools [124]. An incarcerated hernia may also present as an irreducible groin or abdominal wall bulge, often associated with pain, tenderness, and overlying skin erythema or discoloration. Ultrasound can be used to confirm herniated contents, assess reducibility, and evaluate perfusion when strangulation is suspected. Emergent surgical exploration is required to repair the hernia and salvage the ischemic bowel prior to the onset of irreversible necrosis, failing which, outcomes may be fatal [117,118].

### 2.16. Food Protein-Induced Enterocolitis Syndrome (FPIES)

Food protein-induced enterocolitis syndrome (FPIES) is a non-IgE mediated gastrointestinal food hypersensitivity disorder that most commonly occurs in response to cow’s milk, soy, or rice proteins. Unlike IgE-mediated food allergy, symptoms typically develop 2–6 h after antigen exposure and include profuse vomiting, pallor, lethargy, diarrhea, and in severe cases, shock. FPIES usually presents during the first months of life, particularly in formula-fed infants, and may closely resemble NEC. In a cohort of infants with presumed NEC, FPIES was identified in 6.7% of term and 44.7% of preterm infants, highlighting the potential for diagnostic confusion [125].

The pathogenesis of FPIES is incompletely understood but appears to involve aberrant activation of the innate immune system, with a T-helper 2-predominant inflammatory response resulting in an imbalance between increased pro-inflammatory tumor necrosis factor-alpha (TNF-α) secretion and a reduced anti-inflammatory transforming growth factor-beta (TGF-β) response [126,127,128]. This, in turn, leads to increased intestinal permeability and gastrointestinal inflammation. Clinical manifestations range from an acute episode following intermittent exposure, to a chronic form characterized by persistent antigen exposure, failure to thrive, and poor weight gain [95,125,127,129,130].

Distinguishing features from NEC include leucocytosis with eosinophilia, thrombocytosis, and hypoalbuminemia, whereas leucopenia and thrombocytopenia are more commonly observed in NEC [125,131]. Imaging may also aid differentiation, with pneumatosis intestinalis and portal venous gas in FPIES being more localized and resolving within 48 h, while those in NEC tend to be more extensive and persistent as depicted in Table 4 [132]. Reduced bowel motility beyond the affected segment also favors NEC [132]. Diagnosis is based on established international consensus criteria [133], and treatment consists of elimination of the offending food antigen, most commonly through the use of extensively hydrolyzed or amino acid-based formulas, or maternal dietary exclusion in breastfed infants.

## 3. Diagnostic Conundrum

For surgical NEC, a definitive diagnosis is established through intraoperative gross examination, histopathological evaluation of resected bowel, or intestinal findings at autopsy. Minimally invasive tissue sampling may be considered as an alternative to conventional diagnostic autopsy to establish a confirmed tissue diagnosis [134]. In cases managed medically, a definitive diagnosis of NEC may be based on unequivocal clinical confirmation by a neonatologist, incorporating the overall clinical, laboratory, and radiological presentation. The Vermont Oxford Network (VON) 2026 manual of operations defines NEC based on clinical and radiographic criteria, which have been derived from Bell’s staging, or alternatively via examination at the time of surgery or autopsy [135]. The neonate must have at least one clinical and one radiological sign (Appendix A).

However, a number of NEC cases progress to perforation without developing overt clinical signs or early radiological signs, leading to missed opportunities for early intervention. Similarly, cases of SIP may present with abdominal distension and pneumoperitoneum, meeting criteria for definite NEC. FPIES was noted to have pneumatosis and portal venous gas in almost half of the cases [132], with another study showing a higher incidence of pneumatosis in FPIES compared with NEC [125].

Similarly, several of the above differentials, such as volvulus with or without malrotation, intussusception, and neonatal appendicitis, may be misdiagnosed as NEC, leading to a delay in surgical intervention and, potentially, short bowel syndrome. Additionally, HAEC and CHD-associated colitis have a similar pathophysiology to NEC with regards to ischemia, inflammation, and bacterial translocation, with similar management strategies, leading to overestimation of NEC cases.

## 4. Future Perspectives

Emerging non-invasive biomarkers of epithelial injury and inflammation, such as intestinal fatty acid-binding protein (I-FABP) and fetal calprotectin, interpreted alongside cytokine signatures such as interleukin (IL)-6, IL-8, and TGF-β, may support earlier recognition of NEC before definitive clinical patterns are fully established [136]. Metabolomic and oxidative-stress profiling may further refine the diagnosis by capturing downstream metabolic and redox injury. Dried blood spot metabolomics and recent oxidative stress mapping have identified candidate signatures that are pending prospective validation [137]. Integrated multi-omics approaches that combine genomics, transcriptomics, proteomics, and metabolomics are especially attractive because they may link susceptibility, active mucosal injury, and evolving host-microbiome responses within a single diagnostic framework, thereby improving early detection beyond a single marker [138].

Given the lack of sensitive and specific biomarkers, clinicians have no choice but to rely on clinical and radiographic features to diagnose NEC. The use of artificial intelligence to create predictive models using machine learning algorithms sounds promising but is hindered by the lack of a large dataset with high-quality data due to the low incidence of NEC, in addition to data heterogeneity, phenotypic ambiguity, and the “black box” problem [139]. The “black box” dilemma refers to the difficulty for clinicians in understanding how the algorithm came to a particular prediction and the various factors influencing the decision. Small sample sizes and a diverse geographical distribution make training of models and their validation challenging across different demographic profiles. Phenotypic variation in clinical presentation and the large overlap with other similar conditions increase the difficulty in identifying diagnostic patterns of the disease. Ethical and legal issues in data access and sharing compound the challenge of creating predictive models in rare diseases. Bias is another major issue in machine learning algorithms due to the predominance of a particular ethnicity in any geographical location. Therefore, global collaborative efforts between high-volume perinatal and neonatal surgical units, in addition to the involvement of data scientists and artificial intelligence experts, will be the key to overcoming these hurdles and improving the diagnostic accuracy of NEC.

## 5. Conclusions

NEC lacks a single pathognomonic finding, and its clinical, laboratory, and radiographic features overlap substantially with those of other neonatal conditions, including spontaneous intestinal perforation, food protein-induced enterocolitis syndrome, Hirschsprung-associated enterocolitis, CHD-associated colitis, and surgical mimics such as volvulus and intussusception. This overlap contributes to both over- and under-diagnosis of NEC, with implications for management and research. A high index of suspicion for these mimics, as guided by gestational age, timing of onset, and associated risk factors, remains essential. Until validated biomarkers or diagnostic algorithms emerge, accurate discrimination will continue to rely on careful integration of clinical, laboratory, and radiographic findings, supported by awareness of the key differentials outlined in this review.

## Figures and Tables

**Table 1:** Differential diagnoses of NEC which are more common in preterm and term infants.

Preterm Infants	Term Infants
Common	Common
- Spontaneous Intestinal Perforation	- Cow’s Milk Protein Allergy
- Sepsis-associated ileus	- Food protein-induced enterocolitis syndrome
- Anal Fissure	- Hirschsprung-associated enterocolitis
- Dysmotility of Prematurity	- Congenital Heart Disease-associated necrotizing enterocolitis
Rare	Rare
- Malrotation with or without volvulus	- Sepsis-associated ileus
- Cow’s Milk Protein Allergy	- Malrotation with volvulus
- Hirschsprung-associated enterocolitis	- Anal fissure
- Viral enterocolitis	- Viral enterocolitis
- Congenital Heart Disease-associated necrotizing enterocolitis	- Intussusception
- Intussusception	- Neonatal appendicitis
- Neonatal appendicitis	- Incarcerated inguinal hernia
- Meconium obstruction of prematurity	
- Acute mesenteric ischemia	

**Table 2:** Risk factors of differential diagnoses of NEC.

Diagnosis	Risk Factors
Spontaneous Intestinal Perforation	Antenatal - Extreme prematurity [18,19,20] - Maternal chorioamnionitis [21] - Infection—*Candida*, *Staphylococcus epidermidis* [22] - Pre-eclampsia [22] - Twin pregnancy [22] - Fetal growth restriction [22] - Oligohydramnios [22] - Maternal medication—glucocorticoids, indomethacin and magnesium sulphate [22] - Prenatal indomethacin exposure for tocolysis with postnatal early steroids [23] - Velamentous cord insertion [24] Postnatal - Drugs—vasopressors, indomethacin, early postnatal steroids [20,25,26,27,28] - Early prophylactic indomethacin after recent (within 7 days before birth) antenatal steroids [29,30] - No, or minimal, feed volumes prior to perforation [18,22] - Systemic candidiasis [31]
Dysmotility of prematurity	- Extreme prematurity [32] - Very low birth weight [32] - Use of caffeine citrate [32] - Formula feeding [32]
Sepsis-associated ileus	- Systemic inflammation [33]
Volvulus with malrotation	- Fetal growth restriction [34] - Multiple congenital anomalies [35]
Volvulus without malrotation	- Extreme prematurity [34,36,37] - Very low birth weight [34,38] - Small for gestational age [34] - Female sex [34,37] - Non-invasive positive pressure ventilation [34,39] - Pro-peristaltic maneuvers such as intensive abdominal massage and pelvic rotation [37,38]
Cow’s Milk Protein Allergy	- Family history of allergic disorders [40] - Genetic predisposition [41] - Caesarean section [42] - Formula-fed [43]
Hirschsprung-associated enterocolitis	- Anastomotic stenosis [44] - Length of aganglionic segment >30 cms [44] - Pre-operative hypoproteinemia [44] - Down syndrome [44]
Cardiogenic necrotizing enterocolitis	- Prematurity [45] - Very low birth weight [46] - Presence of shock [47,48] - Cyanotic heart disease—hypoplastic left heart syndrome and truncus arteriosus [48,49], single-ventricle heart defect [50] - Need for prostaglandin infusion [47] - Higher doses of prostaglandin >50 ng/kg/min [48] - Red blood cell transfusion [51]
Intussusception	- Meckel’s diverticulum [52] - Intrauterine fetal compromise [53] - Acquired cytomegalovirus infection [54] - Duplication cyst [55]
Neonatal appendicitis	- Prematurity [56] - Male sex [57,58] - Hirschsprung disease [59] - Perinatal asphyxia [58] - Prolonged rupture of membranes or chorioamnionitis [58] - Congenital heart disease [58] - Maternal smoking [58]
Meconium obstruction	- Prematurity [60,61,62] - Very low birth weight [63,64] - Maternal hypertensive disorder [60,61,63,64] - Maternal treatment with magnesium sulphate [60,61] - Fetal growth restriction [61] - Fetal distress [64]
Acute mesenteric ischemia	- Occlusive—prothrombotic state, umbilical arterial catheter, dehydration [65] - Non-occlusive—vasopressor use, post-cardiac arrest, hypotension, hypovolemia, dehydration, sepsis [65]

**Table 3:** Risk factors and mechanism of spontaneous intestinal perforation.

Risk Factors	Mechanism
Extreme prematurity [70]	Intestinal vulnerability—nitric oxide depletion, decreased transforming growth factor-alpha (TGF-α)
Maternal chorioamnionitis [21]	Fetal inflammatory response
Invasive infections associated with *Canadia*, *Staphylococcus epidermidis* [22]	Cytokine-mediated acute inflammation
Maternal conditions: [22] Pre-eclampsia, twin pregnancy, fetal growth restriction, oligohydramnios Maternal Drugs: Glucocorticoids, cocaine, magnesium sulphate, indomethacin [23]	Fetal hypoxia
Velamentous cord insertion [24]	Fetal ischemia
Vasopressors, early prophylactic indomethacin, early postnatal steroids [20,25,26,27,28,29,30]	Mesenteric ischemia

**Table 4:** Imaging findings in food protein-induced enterocolitis syndrome.

Imaging	FPIES
Abdominal ultrasound findings [132]	Bowel wall thickening (90%)
	PI and PVG (42%)
	Reduced motility in lesion area
	Ascites (19%)
	Hypoechoic change in gallbladder wall (2%)

PI: pneumatosis intestinalis, PVG: portal venous gas.

## Data Availability

No new data were created or analyzed in this study. Data sharing is not applicable to this article.

## Abbreviations

CHD	Congenital heart disease
CMPA	Cow’s milk protein allergy
ELBW	Extremely low birth weight
FPIES	Food protein-induced enterocolitis syndrome
GA	Gestational age
HAEC	Hirschsprung-associated enterocolitis
HD	Hirschsprung disease
IgE	Immunoglobulin E
IL	Interleukin
NEC	Necrotizing enterocolitis
NOS	Nitric oxide synthetase
OR	Odds ratio
PI	pneumatosis intestinalis
PMA	Post-menstrual age
PNA	Post-natal age
PVG	Portal venous gas
RR	Relative risk
SIP	Spontaneous intestinal perforation
TGF-β	Transforming growth factor-beta
TNF-α	Tumor necrosis factor-alpha
VLBW	Very low birth weight
VON	Vermont Oxford Network
VWM	Volvulus without malrotation

## Appendix A. Staging of NEC

**Table A1:** Diagnosis of Necrotizing enterocolitis.

Modified Bell’s Staging [16]			
	Local Signs	Systemic Signs	Radiological Features
Modified Bell Ia	Increased gastric residuals, mild abdominal distension, emesis	Temperature instability, apnea, bradycardia, lethargy	Normal or mild intestinal dilatation, mild ileus
Modified Bell Ib	Same as Ia plus grossly bloody stool	Same as Ia	Same as Ia
Modified Bell IIa	Same as Stage I with absent bowel sounds ± abdominal tenderness	Same as Ia	Intestinal dilatation, ileus, pneumatosis intestinalis
Modified Bell IIb	Same as IIa plus definite abdominal tenderness ± cellulitis/right lower quadrant mass	Same as IIa plus mild metabolic acidosis and thrombocytopenia	Same as IIa plus portal venous gas ± ascites
Modified Bell IIIa	Same as IIb with signs of impending respiratory failure due to abdominal distension	Hypotension, bradycardia, apnea, combined metabolic and respiratory acidosis, DIC, neutropenia	Definite ascites without pneumoperitoneum
Modified Bell IIIb	Same as IIIa plus evidence of perforation	Same as IIIa	Pneumoperitoneum (evidence of intestinal perforation)
**VON criteria** [135]			
Any one clinical feature - bilious gastric aspirate or emesis - abdominal distension or discoloration - occult or gross blood in stool without a fissure			
Any one radiological feature - pneumatosis intestinalis - hepato-biliary gas - pneumoperitoneum			

## References

[1] Alsaied A., Islam N., Thalib L. (2020). Global incidence of Necrotizing Enterocolitis: A systematic review and Meta-analysis. BMC Pediatr..

[2] Han S.M., Hong C.R., Knell J., Edwards E.M., Morrow K.A., Soll R.F., Modi B.P., Horbar J.D., Jaksic T. (2020). Trends in incidence and outcomes of necrotizing enterocolitis over the last 12 years: A multicenter cohort analysis. J. Pediatr. Surg..

[3] Walsh M.C., Kliegman R.M., Hack M. (1989). Severity of necrotizing enterocolitis: Influence on outcome at 2 years of age. Pediatrics.

[4] Pitt J.B., Linton S., Zeineddin S., Carter M., Ghomrawi H., Abdullah F. (2023). Mortality of necrotizing enterocolitis does not vary across tertiary care children’s hospitals. J. Pediatr. Surg. Open.

[5] Hull M.A., Fisher J.G., Gutierrez I.M., Jones B.A., Kang K.H., Kenny M., Zurakowski D., Modi B.P., Horbar J.D., Jaksic T. (2014). Mortality and management of surgical necrotizing enterocolitis in very low birth weight neonates: A prospective cohort study. J. Am. Coll. Surg..

[6] Blakely M.L., Gupta H., Lally K.P. (2008). Surgical management of necrotizing enterocolitis and isolated intestinal perforation in premature neonates. Semin. Perinatol..

[7] Schulzke S.M., Deshpande G.C., Patole S.K. (2007). Neurodevelopmental Outcomes of Very Low-Birth-Weight Infants With Necrotizing Enterocolitis: A Systematic Review of Observational Studies. Arch. Pediatr. Adolesc. Med..

[8] Han S.M., Knell J., Henry O., Riley H., Hong C.R., Staffa S.J., Modi B.P., Jaksic T. (2020). Long-term outcomes of severe surgical necrotizing enterocolitis. J. Pediatr. Surg..

[9] Canvasser J., Patel R.M., Pryor E., Green L., Hintz S.R., Fagan M., Harrison J.D. (2023). Long-term outcomes and life-impacts of necrotizing enterocolitis: A survey of survivors and parents. Semin. Perinatol..

[10] Wang Y., Liu S., Lu M., Huang T., Huang L. (2024). Neurodevelopmental outcomes of preterm with necrotizing enterocolitis: A systematic review and meta-analysis. Eur. J. Pediatr..

[11] Okten E.I., Frankl M., Wu S., Gamaty H., Thompson H., Yardley I.E. (2024). Factors affecting neurodevelopmental outcome following surgical necrotising enterocolitis: A systematic review. Pediatr. Surg. Int..

[12] Lamireau N., Greiner E., Hascoët J.-M. (2023). Risk factors associated with necrotizing enterocolitis in preterm infants: A case–control study. Arch. Pediatr..

[13] Vaks Y., Birnie K.L., Carmichael S.L., Hernandez-Boussard T., Benitz W.E. (2018). Temporal Relationship of Onset of Necrotizing Enterocolitis and Introduction of Enteric Feedings and Powdered Milk Fortifier. Am. J. Perinatol..

[14] Gordon P.V., Clark R., Swanson J.R., Spitzer A. (2014). Can a national dataset generate a nomogram for necrotizing enterocolitis onset?. J. Perinatol..

[15] Bell M.J., Ternberg J.L., Feigin R.D., Keating J.P., Marshall R., Barton L., Brotherton T. (1978). Neonatal necrotizing enterocolitis. Therapeutic decisions based upon clinical staging. Ann. Surg..

[16] Walsh M.C., Kliegman R.M. (1986). Necrotizing enterocolitis: Treatment based on staging criteria. Pediatr. Clin. N. Am..

[17] Challis P., Larsson L., Stoltz Sjöström E., Serenius F., Domellöf M., Elfvin A. (2019). Validation of the diagnosis of necrotising enterocolitis in a Swedish population-based observational study. Acta Paediatr..

[18] Meyer C.L., Payne N.R., Roback S.A. (1991). Spontaneous, isolated intestinal perforations in neonates with birth weight less than 1,000 g not associated with necrotizing enterocolitis. J. Pediatr. Surg..

[19] Holland A.J., Shun A., Martin H.C., Cooke-Yarborough C., Holland J. (2003). Small bowel perforation in the premature neonate: Congenital or acquired?. Pediatr. Surg. Int..

[20] Thakkar P.V., Sutton K.F., Detwiler C.-A.B., Henegar J.G., Narayan J.R., Perez-Romero M., Strausser C.M., Clark R.H., Benjamin D.K., Zimmerman K.O. (2024). Risk factors and epidemiology of spontaneous intestinal perforation among infants born at 22-24 weeks’ gestational age. J. Perinatol..

[21] Ragouilliaux C.J., Keeney S.E., Hawkins H.K., Rowen J.L. (2007). Maternal factors in extremely low birth weight infants who develop spontaneous intestinal perforation. Pediatrics.

[22] Gordon P.V. (2009). Understanding intestinal vulnerability to perforation in the extremely low birth weight infant. Pediatr. Res..

[23] Mantle A., Yang M.J., Judkins A., Chanthavong I., Yoder B.A., Chan B. (2024). Association of Intrapartum Drugs with Spontaneous Intestinal Perforation: A Single-Center Retrospective Review. Am. J. Perinatol..

[24] Nakajima Y., Masaoka N., Yamamoto T. (2011). Obstetrical risk factors for focal intestinal perforation in very low birth weight infants. J. Perinat. Med..

[25] Gordon P., Rutledge J., Sawin R., Thomas S., Woodrum D. (1999). Early Postnatal Dexamethasone Increases the Risk of Focal Small Bowel Perforation in Extremely Low Birth Weight Infants. J. Perinatol..

[26] Attridge J.T., Clark R., Walker M.W., Gordon P.V. (2006). New insights into spontaneous intestinal perforation using a national data set: (1) SIP is associated with early indomethacin exposure. J. Perinatol..

[27] Hwang H., Murphy J.J., Gow K.W., Magee J.F., Bekhit E., Jamieson D. (2003). Are localized intestinal perforations distinct from necrotizing enterocolitis?. J. Pediatr. Surg..

[28] Watterberg K.L., Gerdes J.S., Cole C.H., Aucott S.W., Thilo E.H., Mammel M.C., Couser R.J., Garland J.S., Rozycki H.J., Leach C.L. (2004). Prophylaxis of early adrenal insufficiency to prevent bronchopulmonary dysplasia: A multicenter trial. Pediatrics.

[29] Kandraju H., Kanungo J., Lee K.-S., Daspal S., Adie M.A., Dorling J., Ye X.Y., Lee S.K., Shah P.S., Beltempo M. (2021). Association of Co-Exposure of Antenatal Steroid and Prophylactic Indomethacin with Spontaneous Intestinal Perforation. J. Pediatr..

[30] Arnautovic T.I., Longo J.L., Trail-Burns E.J., Tucker R., Keszler M., Laptook A.R. (2021). Antenatal Risk Factors Associated with Spontaneous Intestinal Perforation in Preterm Infants Receiving Postnatal Indomethacin. J. Pediatr..

[31] Adderson E.E., Pappin A., Pavia A.T. (1998). Spontaneous intestinal perforation in premature infants: A distinct clinical entity associated with systemic candidiasis. J. Pediatr. Surg..

[32] Huang X., Chen Q., Peng W. (2018). Clinical characteristics and risk factors for feeding intolerance in preterm infants. Zhong Nan DA Xue Xue Bao YI Xue Ban.

[33] Basu S. (2015). Neonatal sepsis: The gut connection. Eur. J. Clin. Microbiol. Infect. Dis..

[34] Maas C., Hammer S., Kirschner H.J., Yarkin Y., Poets C.F., Franz A.R. (2014). Late-onset volvulus without malrotation in extremely preterm infants—A case-control-study. BMC Pediatr..

[35] Human M.J., Tshifularo N., Hawu Y. (2022). Intestinal malrotation with multiple congenital anomalies. J. Pediatr. Surg. Case Rep..

[36] Kargl S., Wagner O., Pumberger W. (2015). Volvulus without malposition—A single-center experience. J. Surg. Res..

[37] Billiemaz K., Varlet F., Patural H., Rayet I., Tardieu D., Lavocat M.P., Teyssier G. (2001). Intestinal volvulus in extremely premature infants. Arch. Pediatr..

[38] Zweifel N., Meuli M., Subotic U., Moehrlen U., Mazzone L., Arlettaz R. (2013). Manufactured volvulus. Eur. J. Pediatr. Surg..

[39] Drewett M., Burge D.M. (2009). Late-onset volvulus without malrotation in preterm infants. J. Pediatr. Surg..

[40] Kalach N., Bellaïche M., Elias-Billon I., Dupont C. (2019). Family history of atopy in infants with cow’s milk protein allergy: A French population-based study. Arch. Pediatr..

[41] Hou L., Ma Z., Chao S., Li Z., Zhang Y., Su Q., Zhang J., Wu W., Zeng C., Huang S. (2022). Genetic susceptibility to cow’s milk allergy in Chinese children. Asia Pac. J. Clin. Nutr..

[42] Jaiswal L., Worku M. (2022). Recent perspective on cow’s milk allergy and dairy nutrition. Crit. Rev. Food Sci. Nutr..

[43] Sardecka I., Łoś-Rycharska E., Ludwig H., Gawryjołek J., Krogulska A. (2018). Early risk factors for cow’s milk allergy in children in the first year of life. Allergy Asthma Proc..

[44] Zhang X.M., Sun D., Xu Q., Liu H., Li Y., Wang D., Wang J., Zhang Q., Hou P.M., Mu W.M. (2023). Risk factors for Hirschsprung disease-associated enterocolitis: A systematic review and meta-analysis. Int. J. Surg..

[45] Steurer M.A., Baer R.J., Keller R.L., Oltman S., Chambers C.D., Norton M.E., Peyvandi S., Rand L., Rajagopal S., Ryckman K.K. (2017). Gestational Age and Outcomes in Critical Congenital Heart Disease. Pediatrics.

[46] Fisher J.G., Bairdain S., Sparks E.A., Khan F.A., Archer J.M., Kenny M., Edwards E.M., Soll R.F., Modi B.P., Yeager S. (2015). Serious congenital heart disease and necrotizing enterocolitis in very low birth weight neonates. J. Am. Coll. Surg..

[47] Leung M.P., Chau K.T., Hui P.W., Tam A.Y., Chan F.L., Lai C.L., Yeung C.Y. (1988). Necrotizing enterocolitis in neonates with symptomatic congenital heart disease. J. Pediatr..

[48] McElhinney D.B., Hedrick H.L., Bush D.M., Pereira G.R., Stafford P.W., Gaynor J.W., Spray T.L., Wernovsky G. (2000). Necrotizing enterocolitis in neonates with congenital heart disease: Risk factors and outcomes. Pediatrics.

[49] Lau P.E., Cruz S.M., Ocampo E.C., Nuthakki S., Style C.C., Lee T.C., Wesson D.E., Olutoye O.O. (2018). Necrotizing enterocolitis in patients with congenital heart disease: A single center experience. J. Pediatr. Surg..

[50] Becker K.C., Hornik C.P., Cotten C.M., Clark R.H., Hill K.D., Lenfestey R.W., Smith P.B. (2015). Necrotizing enterocolitis in infants with ductal-dependent congenital heart disease. Am. J. Perinatol..

[51] Baxi A.C., Josephson C.D., Iannucci G.J., Mahle W.T. (2014). Necrotizing enterocolitis in infants with congenital heart disease: The role of red blood cell transfusions. Pediatr. Cardiol..

[52] Prakash A., Doshi B., Singh S., Vyas T., Jain A. (2015). Intussusception in a premature neonate: A rare and often misdiagnosed clinical entity. Afr. J. Paediatr. Surg..

[53] Hirokawa S., Uotani H., Yoshida T., Tsukada K. (2001). Ileoileal intussusception and ileal stricture associated with necrotizing enterocolitis in a premature infant: Report of a case. Surg. Today.

[54] Naito S., Fukushima S., Ioroi T., Sakata C., Kurokawa D., Egawa T., Tsuruno Y., Okamoto M., Fukuzawa H., Kugo M. (2024). A Case of an Extremely Preterm Infant with Intussusception Triggered by Acquired Cytomegalovirus Infection. Kobe J. Med. Sci..

[55] Jung J.H., Park J.Y., Park S.-H. (2021). Small Bowel Intussusception in a Preterm Infant. Perinatology.

[56] Liu Y., Yu X., Zhang G., Xie C., Li Y., Mu P., Chen S., Chen Y., Huang S. (2023). Preterm Birth and Infantile Appendicitis. Pediatrics.

[57] Karaman A., Cavuşoğlu Y.H., Karaman I., Cakmak O. (2003). Seven cases of neonatal appendicitis with a review of the English language literature of the last century. Pediatr. Surg. Int..

[58] Raveenthiran V. (2015). Neonatal Appendicitis (Part 1): A Review of 52 cases with Abdominal Manifestation. J. Neonatal Surg..

[59] Martin L.W., Perrin E.V. (1967). Neonatal perforation of the appendix in association with Hirschsprung’s disease. Ann. Surg..

[60] Krasna I.H., Rosenfeld D., Salerno P. (1996). Is it necrotizing enterocolitis, microcolon of prematurity, or delayed meconium plug? A dilemma in the tiny premature infant. J. Pediatr. Surg..

[61] Greenholz S.K., Perez C., Wesley J.R., Marr C.C. (1996). Meconium obstruction in markedly premature infant. J. Pediatr. Surg..

[62] Emil S., Nguyen T., Sills J., Padilla G. (2004). Meconium obstruction in extremely low-birth-weight neonates: Guidelines for diagnosis and management. J. Pediatr. Surg..

[63] Garza-Cox S., Keeney S.E., Angel C.A., Thompson L.L., Swischuk L.E. (2004). Meconium obstruction in the very low birth weight premature infant. Pediatrics.

[64] Byun J., Han J.-W., Youn J.K., Yang H.-B., Shin S.H., Kim E.-K., Kim H.-Y., Jung S.-E. (2020). Risk factors of meconium-related ileus in very low birth weight infants: Patients-control study. Sci. Rep..

[65] Chan S.B., Ng B.W., Chieng C.H. (2024). Acute mesenteric ischaemia: A rare complication mimicking necrotising enterocolitis in a premature infant. BMJ Case Rep..

[66] Pumberger W., Mayr M., Kohlhauser C., Weninger M. (2002). Spontaneous localized intestinal perforation in very-low-birth-weight infants: A distinct clinical entity different from necrotizing enterocolitis. J. Am. Coll. Surg..

[67] Kawase Y., Ishii T., Arai H., Uga N. (2006). Gastrointestinal perforation in very low-birthweight infants. Pediatr. Int..

[68] Fatemizadeh R., Mandal S., Gollins L., Shah S., Premkumar M., Hair A. (2021). Incidence of spontaneous intestinal perforations exceeds necrotizing enterocolitis in extremely low birth weight infants fed an exclusive human milk-based diet: A single center experience. J. Pediatr. Surg..

[69] Mao W., Network O.B.O.T.C.N., Jiang S., Shen C., Zhu H., Wang Y., Zeng L., Mi H., Long Y., Li Z. (2024). Spontaneous intestinal perforation among very preterm infants in China: A multicenter cohort study. Transl. Pediatr..

[70] Elgendy M.M., Othman H.F., Heis F., Qattea I., Aly H. (2021). Spontaneous intestinal perforation in premature infants: A national study. J. Perinatol..

[71] Berrington J.E., Embleton N.D. (2021). Time of Onset of Necrotizing Enterocolitis and Focal Perforation in Preterm Infants: Impact on Clinical, Surgical, and Histological Features. Front. Pediatr..

[72] Buchheit J.Q., Stewart D.L. (1994). Clinical Comparison of Localized Intestinal Perforation and Necrotizing Enterocolitis in Neonates. Pediatrics.

[73] Olaloye O., Swatski M., Konnikova L. (2020). Role of Nutrition in Prevention of Neonatal Spontaneous Intestinal Perforation and Its Complications: A Systematic Review. Nutrients.

[74] Gordon P.V., Herman A.C., Marcinkiewicz M., Gaston B.M., Laubach V.E., Aschner J.L. (2007). A neonatal mouse model of intestinal perforation: Investigating the harmful synergism between glucocorticoids and indomethacin. J. Pediatr. Gastroenterol. Nutr..

[75] Chen Y.-H., Chung H.-W., Chen H.-L., Chang Y.-T. (2023). Clinical significance of black-bluish discoloration of the abdominal wall in the newborn. Formos. J. Surg..

[76] Fischer A., Vachon L., Durand M., Cayabyab R.G. (2015). Ultrasound to diagnose spontaneous intestinal perforation in infants weighing ⩽ 1000 g at birth. J. Perinatol..

[77] Chen Y., Chang K.T.E., Lian D.W.Q., Lu H., Roy S., Laksmi N.K., Low Y., Krishnaswamy G., Pierro A., Ong C.C.P. (2016). The role of ischemia in necrotizing enterocolitis. J. Pediatr. Surg..

[78] Miserez M., Barten S., Geboes K., Naulaers G., Devlieger H., Penninckx F. (2003). Surgical therapy and histological abnormalities in functional isolated small bowel obstruction and idiopathic gastrointestinal perforation in the very low birth weight infant. World J. Surg..

[79] Gordon P.V., Swanson J.R., Attridge J.T., Clark R. (2007). Emerging trends in acquired neonatal intestinal disease: Is it time to abandon Bell’s criteria?. J. Perinatol..

[80] Wadhawan R., Oh W., Hintz S.R., Blakely M.L., Das A., Bell E.F., Saha S., Laptook A.R., Shankaran S., Stoll B.J. (2014). Neurodevelopmental outcomes of extremely low birth weight infants with spontaneous intestinal perforation or surgical necrotizing enterocolitis. J. Perinatol..

[81] Humberg A., Spiegler J., Fortmann M.I., Zemlin M., Marissen J., Swoboda I., Rausch T.K., Herting E., Göpel W., Härtel C. (2020). Surgical necrotizing enterocolitis but not spontaneous intestinal perforation is associated with adverse neurological outcome at school age. Sci. Rep..

[82] Vaidya R., Yi J.X., O’sHea T.M., Jensen E.T., Joseph R.M., Shenberger J., Gogcu S., Wagner K., Msall M.E., Thompson A.L. (2022). Long-Term Outcome of Necrotizing Enterocolitis and Spontaneous Intestinal Perforation. Pediatrics.

[83] Culbreath K., Keefe G., Edwards E.M., Morrow K.A., Soll R.F., Jaksic T., Horbar J.D., Modi B.P. (2022). Morbidity associated with laparotomy-confirmed spontaneous intestinal perforation: A prospective multicenter analysis. J. Pediatr. Surg..

[84] Costner B.J., Carter B.S., Wentz S.C., Wills M.L. (2011). Intestinal malrotation in an extremely preterm very low birthweight infant. BMJ Case Rep..

[85] Mishra P.R., Stringer M.D. (2021). Intestinal malrotation in extremely premature infants: A potential trap. Pediatr. Surg. Int..

[86] Nguyen H.N., Navarro O.M., Bloom D.A., Feinstein K.A., Guillerman R.P., Munden M.M., Sammer M.B.K., Silva C.T. (2022). Ultrasound for Midgut Malrotation and Midgut Volvulus: AJR Expert Panel Narrative Review. Am. J. Roentgenol..

[87] Has R., Gunay S. (2002). ‘Whirlpool’ sign in the prenatal diagnosis of intestinal volvulus. Ultrasound Obstet. Gynecol..

[88] Ruiz-Tovar J., Morales V., Sanjuanbenito A., Lobo E., Martinez-Molina E. (2009). Volvulus of the Small Bowel in Adults. Am. Surg..

[89] Vergnes P., Boissinot F., Pontailler J.R., Demarquez J.L., Laloge V., Bondonny J.M. (1989). Primary volvulus of the small intestine without malrotation. Apropos of 7 cases. Ann. Pediatr..

[90] Di Maggio G., De Felice C., Messina M., Biagini G., Tota G., Bracci R. (1997). Intrauterine volvulus without malrotation in a very low-birth-weight preterm infant. Eur. J. Pediatr. Surg..

[91] Raherison R., Grosos C., Lemale J., Blondiaux E., Sabourdin N., Dahan S., Rosenblatt J., Guilbert J., Jouannic J.M., Mitanchez D. (2012). Prenatal intestinal volvulus: A life-threatening event with good long-term outcome. Arch. Pediatr..

[92] Berseth C.L. (1996). Gastrointestinal Motility in the Neonate. Clin. Perinatol..

[93] Neu J. (2007). Gastrointestinal development and meeting the nutritional needs of premature infants23. Am. J. Clin. Nutr..

[94] Cianferoni A., Spergel J.M. (2009). Food Allergy: Review, Classification and Diagnosis. Allergol. Int..

[95] Leonard S.A., Nowak-Węgrzyn A. (2011). Food protein-induced enterocolitis syndrome: An update on natural history and review of management. Ann. Allergy Asthma Immunol..

[96] Florquin M., Eerdekens A. (2023). What is Known About Cow’s Milk Protein Allergy in Preterm Infants?. Breastfeed. Med..

[97] Mazon A., Solera E., Alentado N., Oliver F., Pamies R., Caballero L., Nieto A., Dalmau J. (2008). Frequent IgE sensitization to latex, cow’s milk, and egg in children with short bowel syndrome. Pediatr. Allergy Immunol..

[98] Duess J.W., Hofmann A.D., Puri P. (2014). Prevalence of Hirschsprung’s disease in premature infants: A systematic review. Pediatr. Surg. Int..

[99] Gosain A., Frykman P.K., Cowles R.A., Horton J., Levitt M., Rothstein D.H., Langer J.C., Goldstein A.M. (2017). On behalf of the American Pediatric Surgical Association Hirschsprung Disease Interest Group. Guidelines for the diagnosis and management of Hirschsprung-associated enterocolitis. Pediatr. Surg. Int..

[100] Siano E., Lauriti G., Ceccanti S., Zani A. (2019). Cardiogenic Necrotizing Enterocolitis: A Clinically Distinct Entity from Classical Necrotizing Enterocolitis. Eur. J. Pediatr. Surg..

[101] Carlo W.F., Kimball T.R., Michelfelder E.C., Border W.L. (2007). Persistent diastolic flow reversal in abdominal aortic Doppler-flow profiles is associated with an increased risk of necrotizing enterocolitis in term infants with congenital heart disease. Pediatrics.

[102] Miller T.A., Minich L.L., Lambert L.M., Joss-Moore L., Puchalski M.D. (2014). Abnormal abdominal aorta hemodynamics are associated with necrotizing enterocolitis in infants with hypoplastic left heart syndrome. Pediatr. Cardiol..

[103] Ellis C.L., Bokulich N.A., Kalanetra K.M., Mirmiran M., Elumalai J., Haapanen L., Schegg T., Rutledge J.C., Raff G., Mills D.A. (2013). Probiotic administration in congenital heart disease: A pilot study. J. Perinatol..

[104] Cognata A., Kataria-Hale J., Griffiths P., Maskatia S., Rios D., O’Donnell A., Roddy D.J., Mehollin-Ray A., Hagan J., Placencia J. (2019). Human Milk Use in the Preoperative Period Is Associated with a Lower Risk for Necrotizing Enterocolitis in Neonates with Complex Congenital Heart Disease. J. Pediatr..

[105] Kotb M., Abdelatty M., Rashwan H., AbdelMeguid Y., Elrouby A. (2021). Intussusception in preterm neonates: A systematic review of a rare condition. BMC Pediatr..

[106] Avansino J.R., Bjerke S., Hendrickson M., Stelzner M., Sawin R. (2003). Clinical features and treatment outcome of intussusception in premature neonates. J. Pediatr. Surg..

[107] Qafesha R.M., Sharabati I., Kashbour M., Elbadry M., Eldeeb H., Ayesh B.M., Elganady A., Abdelgalil M.S., Abdulrazzak M., Jobran A.W.M. (2025). Pneumatic versus liquid enema reduction in the management of pediatric intussusception: An updated systematic review and meta-analysis. Ann. Med. Surg..

[108] Lim P.P., Hassan H., Roodzi A.H. (2025). Distinguishing intussusception from necrotizing enterocolitis in a preterm infant: A case report. Pediatr. Med..

[109] Vakrilova L., Georgiev T., Hitrova S., Slancheva B. (2014). Perforated neonatal appendicitis in a preterm newborn. Akush. Ginekol..

[110] Delgado C.A., Sánchez V., Shimabuku R., Cadillo G., Tabuchi M., Durand F. (2021). Neonatal Appendicitis. J. Pediatr. Surg. Case Rep..

[111] Tumen A., Chotai P.N., Williams J.M., Myers-Webb A., Krishnan R., Eubanks J.W. (2017). Neonatal Perforated Appendicitis Attributed to Localized Necrotizing Enterocolitis of the Appendix: A Review. J. Neonatal Surg..

[112] Fascetti-Leon F., Sherwood W. (2017). Neonatal Appendicitis and Incarcerated Inguinal Hernia: Case Report and Review of the Literature. J. Indian Assoc. Pediatr. Surg..

[113] Schwartz K.L., Gilad E., Sigalet D., Yu W., Wong A.L. (2011). Neonatal acute appendicitis: A proposed algorithm for timely diagnosis. J. Pediatr. Surg..

[114] Giacoia G.P., Williams G.P. (1995). Rectal bleeding due to nonspecific colitis in premature infants. South. Med. J..

[115] Bowen A. (1980). Mild form of neonatal necrotizing enterocolitis masked by anal fissures. Radiology.

[116] Reeves P.T., James-Davis L.T., Khan M.A. (2023). Gastrointestinal bleeding in the neonate: Updates on diagnostics, therapeutics, and management. NeoReviews.

[117] Hirata K., Kawahara H., Shiono N., Nishihara M., Kubota A., Nakayama M., Kitajima H. (2015). Mesenteric hernia causing bowel obstruction in very low-birthweight infants. Pediatr. Int..

[118] Kylat R.I. (2017). Internal Hernia Masquerading As Necrotizing Enterocolitis. Front. Pediatr..

[119] Dilek Kurnaz B.C. (2023). Derya Buyukkayhan Enteroscrotal Fistula Following Incarcerated Inguinal Hernia that Presented as NECin a Preterm Baby Mathews. J. Pediatr..

[120] Lim A.M., Yap T.L., Kong J.Y. (2023). Incarcerated hernia with ileal perforation in an extreme preterm infant. BMJ Case Rep..

[121] Prasad G., Dalai R., Dhua A.K., Anand S. (2023). Postoperative necrotising enterocolitis following incarcerated inguinal hernia repair. BMJ Case Rep..

[122] Kumar C., Nagendla M.K., Pothala R., Bathina S.P., Chandrasekharam V.V.S. (2022). Postoperative necrotizing enterocolitis due to incarcerated inguinal hernia in a preterm infant at term age equivalent. J. Pediatr. Surg. Case Rep..

[123] Türkyilmaz Z., Sönmez K., Numanoğlu V., Kale N., Başaklar A.C. (2001). Postoperative necrotizing enterocolitis following incarcerated inguinal hernia repair: Report of a case. Surg. Today.

[124] Lamprinou Z., Kanna E., Skondras I., Sfakiotaki R., Mene J., Achilleos O. (2024). Internal hernia as cause of acute abdomen in a preterm neonate: When necrotizing enterocolitis is not the culprit. Arch. Clin. Cases.

[125] Kim Y.I., Joo J.Y., Jung Y.H., Choi C.W., Kim B.I., Yang H.R. (2022). Differentiation of food protein-induced enterocolitis syndrome misleading to necrotizing enterocolitis. Ann. Allergy Asthma Immunol..

[126] Goswami R., Blazquez A.B., Kosoy R., Rahman A., Nowak-Węgrzyn A., Berin M.C. (2017). Systemic innate immune activation in food protein-induced enterocolitis syndrome. J. Allergy Clin. Immunol..

[127] Nowak-Wegrzyn A., Muraro A. (2009). Food protein-induced enterocolitis syndrome. Curr. Opin. Allergy Clin. Immunol..

[128] Morita H., Nomura I., Shoda T., Unno H., Matsuda A., Saito H., Matsumoto K. (2013). Infants with Food Protein-Induced Enterocolitis Syndrome (FPIES) Can Be Classified Into Two Distinct Subgroups Based On the Presence or Absence of Bloody Stool and Their Antigen-Specific Cytokine Production Profiles. J. Allergy Clin. Immunol..

[129] Boyce J.A., Assa’ad A., Burks A.W., Jones S.M., Sampson H.A., Wood R.A., Plaut M., Cooper S.F., Fenton M.J., Arshad S.H. (2010). Guidelines for the diagnosis and management of food allergy in the United States: Report of the NIAID-sponsored expert panel. J. Allergy Clin. Immunol..

[130] Mehr S., Kakakios A., Frith K., Kemp A.S. (2009). Food protein-induced enterocolitis syndrome: 16-year experience. Pediatrics.

[131] Hwang J.B., Lee S.H., Kang Y.N., Kim S.P., Suh S.I., Kam S. (2007). Indexes of suspicion of typical cow’s milk protein-induced enterocolitis. J. Korean Med. Sci..

[132] Guo Y., Si S., Jia Z., Lv X., Wu H. (2021). Differentiation of food protein-induced enterocolitis syndrome and necrotizing enterocolitis in neonates by abdominal sonography. J. Pediatr..

[133] Nowak-Węgrzyn A., Chehade M., Groetch M.E., Spergel J.M., Wood R.A., Allen K., Atkins D., Bahna S., Barad A.V., Berin C. (2017). International consensus guidelines for the diagnosis and management of food protein-induced enterocolitis syndrome: Executive summary-Workgroup Report of the Adverse Reactions to Foods Committee, American Academy of Allergy, Asthma & Immunology. J. Allergy Clin. Immunol..

[134] Johnson J.M.M.S., Lakshmi V.R., Mathew M. (2026). Standardization of minimally invasive tissue sampling (MITS) technique for intestinal sampling in neonatal autopsies. Forensic Sci. Med. Pathol..

[135] VON (2026). Global Health Neonatal Quality Improvement Database: Data Definitions & Infant Data Sheet. https://vtoxford.zendesk.com/hc/en-us/article_attachments/52401085417363.

[136] Liu S., Liu Y., Lai S., Xie Y., Xiu W., Yang C. (2024). Values of serum intestinal fatty acid-binding protein, fecal calprotectin, and fecal human β-defensin 2 for predicting necrotizing enterocolitis. BMC Pediatr..

[137] Zhang T., Yang S., Li R., Dong R., Zou H. (2024). Dried blood spots-based metabolomic analysis in preterm infants with necrotizing enterocolitis. J. Matern.-Fetal Neonatal Med..

[138] Liu A., Liang T., Zhang R., Zhao S., Kang L., Lei X., Dong W. (2025). Predictive value of biomarkers in neonatal necrotizing enterocolitis. Front. Pediatr..

[139] Mahapatra V., Chandra A., Rehman T., Mitra S., Pradhan P., Kiran A., Agarwalla S.K., Pati S. (2026). Artificial intelligence in the diagnosis of necrotising enterocolitis from abdominal radiographs: A systematic review and meta-analysis. Front. Pediatr..

